# Predicting Pain Response to a Remote Musculoskeletal Care Program for Low Back Pain Management: Development of a Prediction Tool

**DOI:** 10.2196/64806

**Published:** 2024-11-19

**Authors:** Anabela C Areias, Robert G Moulder, Maria Molinos, Dora Janela, Virgílio Bento, Carolina Moreira, Vijay Yanamadala, Steven P Cohen, Fernando Dias Correia, Fabíola Costa

**Affiliations:** 1 Sword Health Inc Draper, UT United States; 2 Institute for Cognitive Science University of Colorado Boulder Boulder, CO United States; 3 Instituto de Ciências Biomédicas Abel Salazar Porto Portugal; 4 Department of Surgery Quinnipiac University Frank H Netter School of Medicine Hamden, CT United States; 5 Department of Neurosurgery Hartford Healthcare Medical Group Westport, CT United States; 6 Department of Anesthesiology and Critical Care Medicine Johns Hopkins School of Medicine Baltimore, MD United States; 7 Department of Physical Medicine and Rehabilitation Johns Hopkins School of Medicine Baltimore, MD United States; 8 Department of Neurology Johns Hopkins School of Medicine Baltimore, MD United States; 9 Department of Psychiatry and Behavioral Sciences Johns Hopkins School of Medicine Baltimore, MD United States; 10 Department of Anesthesiology Uniformed Services University of the Health Sciences Bethesda, MD United States; 11 Department of Physical Medicine and Rehabilitation Uniformed Services University of the Health Sciences Bethesda, MD United States; 12 Neurology Department Centro Hospitalar e Universitário do Porto Porto Portugal

**Keywords:** telerehabilitation, predictive modeling, personalized medicine, rehabilitation, clinical decision support, machine learning, artificial intelligence

## Abstract

**Background:**

Low back pain (LBP) presents with diverse manifestations, necessitating personalized treatment approaches that recognize various phenotypes within the same diagnosis, which could be achieved through precision medicine. Although prediction strategies have been explored, including those employing artificial intelligence (AI), they still lack scalability and real-time capabilities. Digital care programs (DCPs) facilitate seamless data collection through the Internet of Things and cloud storage, creating an ideal environment for developing and implementing an AI predictive tool to assist clinicians in dynamically optimizing treatment.

**Objective:**

This study aims to develop an AI tool that continuously assists physical therapists in predicting an individual’s potential for achieving clinically significant pain relief by the end of the program. A secondary aim was to identify predictors of pain nonresponse to guide treatment adjustments.

**Methods:**

Data collected actively (eg, demographic and clinical information) and passively in real-time (eg, range of motion, exercise performance, and socioeconomic data from public data sources) from 6125 patients enrolled in a remote digital musculoskeletal intervention program were stored in the cloud. Two machine learning techniques, recurrent neural networks (RNNs) and light gradient boosting machine (LightGBM), continuously analyzed session updates up to session 7 to predict the likelihood of achieving significant pain relief at the program end. Model performance was assessed using the area under the receiver operating characteristic curve (ROC-AUC), precision-recall curves, specificity, and sensitivity. Model explainability was assessed using SHapley Additive exPlanations values.

**Results:**

At each session, the model provided a prediction about the potential of being a pain responder, with performance improving over time (*P*<.001). By session 7, the RNN achieved an ROC-AUC of 0.70 (95% CI 0.65-0.71), and the LightGBM achieved an ROC-AUC of 0.71 (95% CI 0.67-0.72). Both models demonstrated high specificity in scenarios prioritizing high precision. The key predictive features were pain-associated domains, exercise performance, motivation, and compliance, informing continuous treatment adjustments to maximize response rates.

**Conclusions:**

This study underscores the potential of an AI predictive tool within a DCP to enhance the management of LBP, supporting physical therapists in redirecting care pathways early and throughout the treatment course. This approach is particularly important for addressing the heterogeneous phenotypes observed in LBP.

**Trial Registration:**

ClinicalTrials.gov NCT04092946; https://clinicaltrials.gov/ct2/show/NCT04092946 and NCT05417685; https://clinicaltrials.gov/ct2/show/NCT05417685

## Introduction

Low back pain (LBP), the leading cause of disability worldwide [1], has a complex and multifaceted etiology [2,3], resulting in varied phenotypes even among individuals with the same diagnosis. Precision medicine supports tailored interventions to address this heterogeneity, with data-driven strategies playing a crucial role [4,5]. These strategies range from simple, pragmatic rules-based methods [6-8] (eg, STARTback, Orebro) to more sophisticated machine learning (ML) tools trained on large data sets [9-11]. In the context of LBP management, ML models have been developed to assist with screening [12-14], assess the probability of a patient transitioning from acute to chronic cases [8,15-18], predict surgical outcomes [19,20], and forecast clinical recovery prognosis [6,7,9-11,21]. Although promising, these models lack sustained, dynamic real-time patient data collection, relying heavily on patient-reported outcome measures (PROMs) and other information that is not easily scalable. This reliance, in turn, limits the ability to deliver real-time predictions throughout treatment or hinders large-scale implementation in real-world settings [9,10].

The emergence of digital care programs (DCPs) as an effective alternative for delivering LBP care [22-24] has facilitated the seamless, automatic collection of abundant data from various sources. This diverse and comprehensive patient data set, which includes passively collected real-time updates of multiple variables via the Internet of Things devices (eg, wearables) enriched with population data, enables the capture of more complex patterns, providing a more accurate representation of patients. These extensive volumes of data can be used to predict outcomes and optimize treatments, with the resulting outcomes feeding back into predictive tools in a virtuous cycle.

Previously, we demonstrated the efficacy of a remote DCP for LBP management [22], which combines exercise, education, and cognitive behavioral therapy under physical therapist (PT) supervision. In this study, we leverage all stored data (both passively and actively collected from patient and public sources) in the cloud portal, actively monitored by the assigned PT, to develop a predictive tool that can assist in optimizing treatment. This study aims to develop an artificial intelligence (AI) tool to assist PTs in predicting an individual’s potential to achieve clinically significant pain relief by the end of the program. Such predictions could enable timely adjustments to the program, increasing the likelihood of success and, in turn, providing data to further refine the tool’s recommendations. As a secondary aim, we sought to identify the predictors of pain nonresponse, as these factors indicate the need for greater attention from PTs. Thus, the overall objective of the study is to develop ML models to predict the likelihood that a patient will achieve clinically significant pain relief by the end of the program, with ongoing updated predictions after each completed session.

## Methods

### Study Design

This secondary analysis utilized data from 2 prospective studies evaluating clinical and engagement-related outcomes in patients with musculoskeletal (MSK) conditions. Data were collected from June 2020 to July 2023. This prognostic study adhered to the TRIPOD-AI (Transparent Reporting of a Multivariable Prediction Model for Individual Prognosis or Diagnosis-AI) reporting guidelines (Table S1 in Multimedia Appendix 1).

### Ethical Considerations

All research was conducted in accordance with the relevant guidelines and regulations set forth by the Declaration of Helsinki. This study received prospective approval from the New England institutional review board (120190313) and Advarra institutional review board (Pro00063337) and was registered on ClinicalTrials.gov (NCT04092946 and NCT05417685) on September 17, 2019, and June 14, 2022, respectively.

All participants provided electronic informed consent to participate in the study, with a waiver of documentation of consent approved by the New England and Advarra institutional review boards.

All collected data underwent a rigorous anonymization process to safeguard the privacy of the individuals involved in the research. The data collection and analysis methods complied with relevant guidelines and regulations. Participants were not offered any form of compensation.

### Population

Beneficiaries of employer health plans from all US states applying to Sword Health’s DCPs were included. The inclusion criteria were as follows: acute or chronic LBP, an average Numerical Pain Rating Scale (NPRS) score of ≥4/10, and at least one NPRS reassessment during the intervention. The exclusion criteria were as follows: (1) health conditions (eg, cardiac or respiratory) that are incompatible with at least 20 minutes of light-to-moderate exercise; (2) cancer-related back pain or current cancer treatment for a non-MSK condition; and (3) serious neurological signs or symptoms, including bowel or bladder dysfunction. All participants provided informed consent.

### Intervention

The DCP consisted of exercise, education, and cognitive behavioral therapy for up to 12 weeks, with a default recommendation of 3 sessions per week, as described elsewhere [22], after enrolling through a dedicated website. Patients completed a baseline condition form that included information on their demographic and clinical characteristics and selected their PT based on their preferences. Subsequently, an onboarding video call was conducted in which the PTs gathered additional medical history and established goals through shared decision-making. Each patient received a Food and Drug Administration–listed class II medical device, which included a mobile app on a dedicated tablet (containing the program) combined with motion tracking, as well as a cloud-based portal that enabled asynchronous remote monitoring and treatment prescription by the PT. A tailored educational program and cognitive behavioral therapy were also provided [25-27]. Bidirectional communication with the PT was facilitated through a secure chat feature on the smartphone app and video calls.

### Outcome

Considering the overarching intervention goal of promoting improvements in pain levels in daily living, pain response was selected as the primary outcome. Pain was assessed using the 11-point NPRS with a 7-day recall period during sessions 9, 18, or 27.

Pain response, defined as at least a 30% reduction in the NPRS by the end of the program, aligns with clinical recommendations from the Initiative on Methods, Measurement, and Pain Assessment in Clinical Trials (IMMPACT) [28]. This definition is supported by a consensus meeting that interpreted the clinical significance of treatment outcomes in clinical trials for chronic pain treatments. Additionally, we included the criterion of concluding the program with an NPRS score equal to or below 3 points (the threshold for mild pain) [29] to account for patients who started with lower pain levels and ended the program with acceptable pain levels. The widespread use of this binary outcome, along with its practicality in real-world settings, supported its inclusion.

### Predictors: Variables Used for Model Development

**Figure 1:**
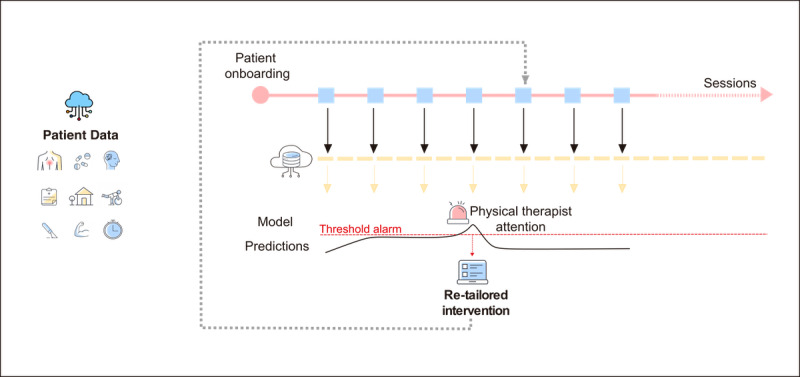
Machine Learning (ML) tool development within the digital care program. All patient data collected both passively and actively regarding demographic and clinical characteristics, range of motion, and session usability, as well as collected from public sources (eg, social deprivation index) are continuously stored from onboarding to the program end, enabling the creation of a data repository within the physical therapists (PTs) cloud portal. At the end of each session, data are processed through an ML model to predict an individual’s potential to achieve clinically significant pain relief at the program end. In the case of the high probability of an unfavorable outcome, an alarm is set in the PT portal for further examination and re-tailoring of the intervention.

The variables used for model development were selected based on existing literature and their clinical relevance to MSK pain. A detailed description of the variable domains and their acceptable ranges can be found in Table 1. The data set variables included (1) demographic characteristics (eg, race and ethnicity, social deprivation index) [30]; (2) baseline clinical presentations (eg, acuity, disability, mental health, productivity, fear-avoidance beliefs, presence of red flags); (3) time series of mean active range of motion (ROM) throughout all repetitions of each exercise during each session; (4) session-related usability (eg, movement errors, number of exercises, sets and repetitions, the time between sessions, session duration); and (5) self-reported fatigue and pain experienced during exercises, measured on a 0-10 scale (Figure 1).

**Table 1:** Features domains included in the study.

Domain and subdomains		Description
**Demographic**		
	Individual characteristics	Comprises information about gender, age, race/ethnicity, body mass index, education level, among others
	Geographical location	Urban or rural location and time zone
	Work characteristics	Employment status and job type
**Socioeconomic**		
	Social deprivation	Assessed by the Social Deprivation Index (score range 0-100) [30]; socially deprived individuals were those with an index ranging from 60 to 100
**Productivity**		
	Participation—work	Assessed by the overall work productivity (score range 0-100), presenteeism (score range 0-100), and absenteeism (score range 0-100) subscales from the Work Productivity Impairment Questionnaire for general health
	Participation—social	Assessed by the non-work–related activities impairment subscale from the Work Productivity Impairment Questionnaire for general health (score range 0-100), and scores of items 8 (range 0-5), 9 (range 0-5), and 10 (range 0-5) of the Oswestry Disability Index
**Clinical**		
	Condition	Includes musculoskeletal condition diagnosis, anatomical pain region, presence of leg symptoms (numbness or tingling), past surgery, among others
	Acuity	Acute (<12 weeks) or chronic (persistent or recurring pain for ≥12 weeks)
	Pain	Pain intensity assessed by the Numerical Pain Rating Scale (score range 0-10)
	Functionality	Assessed by the Oswestry Disability Index (score range 0-100) considering the total score and the individual scores of items 2-7
	Mental health	Assessed by the 7-item General Anxiety Disorder Scale (score range 0-21) and the 9-item Patient Health Questionnaire (score range 0-27), considering the total score and the individual score for each item; features considering moderate and severe depression or anxiety were calculated using the thresholds described in Spitzer et al [31] and Kroenke et al [32]
	Fear-avoidance	Assessed by the Fear-Avoidance Beliefs Questionnaire for Physical Activity (score range 0-24), considering the total score and the individual score for each item
	Surgery intent	Assessed by the question “On a scale of 0 to 100, where 0 is not at all and 100 is extremely interested, how interested are you in undergoing shoulder surgery in the next 12 months?” (score range 0-100)
	Medication consumption	Considers nonopioid analgesic and opioid consumption (yes or no)
	Red flags	Presence/absence of at least one red flag identified during onboard screening, which was cleared before entering the study by a physician (yes or no) [33]
**Prescription**		
	Support level	Includes support-level perception assessed by physical therapist interactions during the intervention
**Exercise Performance**		
	Biomechanics	Active range of motion collected by motion trackers
	Exercise accuracy	Correct and wrong repeats; performance in all prescribed exercises (total correct repeats by total repeats); average stars (0-5)
	Exercise-induced pain acute response	Pain felt during exercise sessions (assessed by the question “How did you feel during your session: my pain during today’s session?” (score range 0-10)
	Exercise-induced fatigue intensity	Self-reported fatigue in response to the question “How did you feel during your session: My fatigue during today’s session?” (score range 0-10)
	Exercise adherence	Session duration time spent on sessions (minutes) and time interval between sessions (days)
**Motivation and Compliance**		
	Motivation	Patient’s individual goals with the intervention and session commitment (through the question “How many 20-minute sessions can you commit to per week?”), among others
	Enrollment aspects	The time span from enrollment to (1) onboarding and (2) session 1
	Days of the week	Days of the week the patient performed the exercise session

All data were automatically stored in the cloud portal. Demographic and clinical characteristics were collected through an onboarding form using validated PROMs. ROM and session usability were passively collected, while fatigue and pain experienced during exercise sessions were recorded at the end of each session.

### Sample Size

Given that there is no established method to calculate sample sizes for prognostic models using ML, we followed the recommended guideline for standard model development, which suggests obtaining 10-20 events per predictor parameter. As we estimated including up to 300 predictor variables in the models, a sample size range of 3000 to 6000 was calculated.

### Missing Data and Data Preparation

The overall missingness was 18.45% (324,385/1,757,875; see Table S2 in Multimedia Appendix 1), comprising the following: 17.07% (300,117/1,757,875) missing completely at random, where the missing data points were attributed to system settings; 0.29% (5169/1,757,875) missing at random, where the missingness could be explained by other variables (eg, specific protocol prescriptions that did not include one of the studied exercises); and 1.09% (19,099/1,757,875) missing not at random, where the reasons for the missing data were related to unobserved factors. Categorical variable imputation was performed by creating a new category labeled “not available,” followed by dummy encoding. Continuous variables were imputed using multiple imputations by chained equations or by assigning a value outside the distribution (eg, –1), depending on the nature of the missingness. Multiple imputations can provide unbiased estimations for data that are missing completely at random and at random. As the rate of missingness for not-at-random data is very small, we used multiple imputation techniques to handle the missing data overall. The cleaned data set included 6125 patients and contained a number of variables ranging from 235 at session 1 to 275 at session 7.

### Analytical Methods

#### Feature Engineering

Feature engineering was utilized to capture meaningful patterns and insights from multiple variables over time, creating new features that represent the underlying relationships and dynamics within the data (as detailed in Figure S1 in Multimedia Appendix 1). A minimum of 4 sessions was required to conduct a longitudinal analysis.

**Figure 2:**
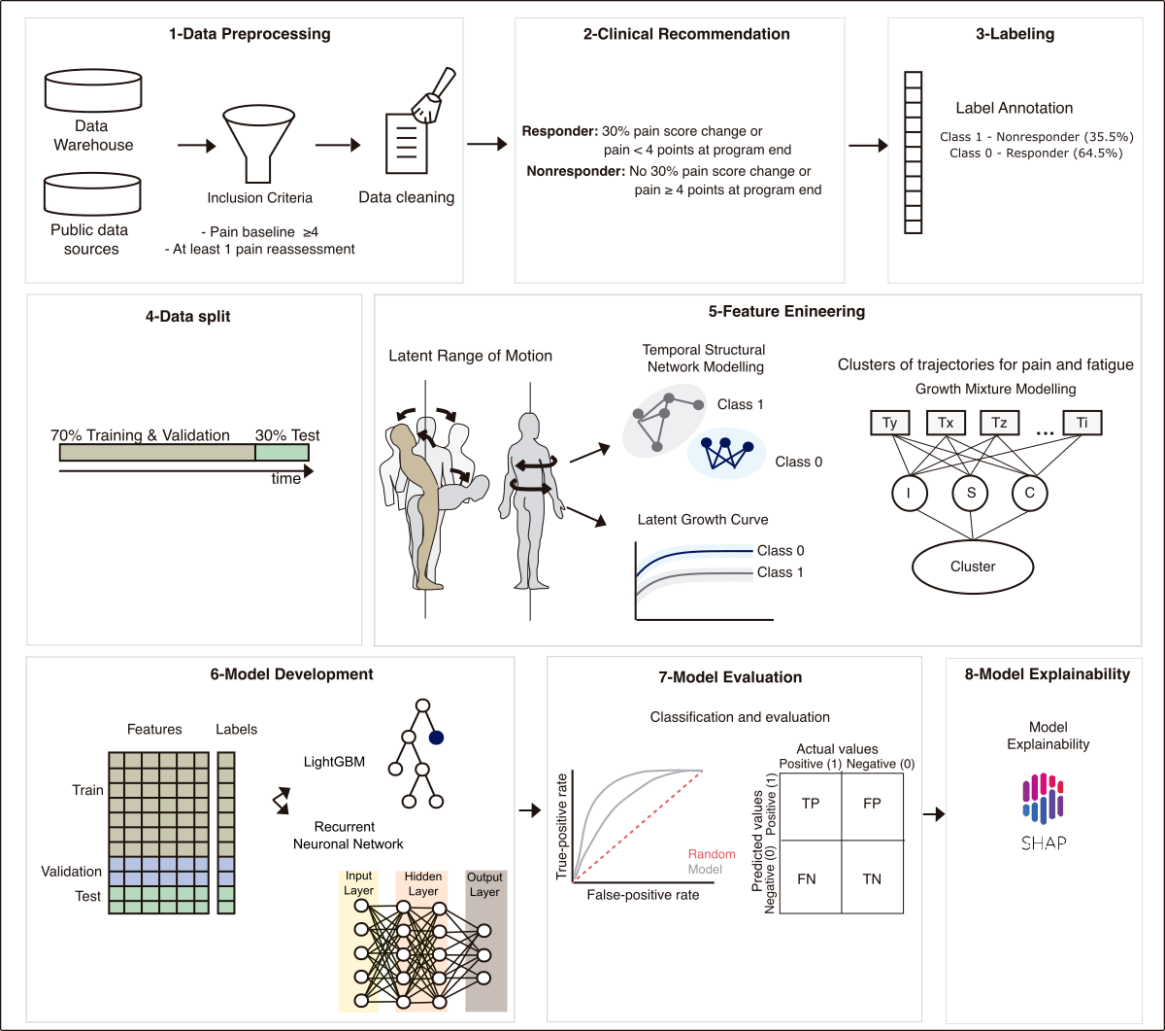
Model development pipeline. Data from patients with low back pain (LBP) who underwent the digital intervention were collected from the data warehouse within a specific time frame. Inclusion criteria: average pain level equal to or greater than 4 on the Numerical Pain Rating Scale (NPRS) at baseline considering a 7-day recall period; and at least one pain reassessment during the intervention. Patient data were preprocessed for the tool. Patients who did not experience a pain (NPRS) reduction of at least 30% or reported pain level higher or equal to 4 at the program end were categorized as nonresponders (labeled as “1” in the data set). Considering the overarching intervention goal of promoting improvement in pain levels, a flag was triggered when patients were likely to be nonresponders to assist PTs’ clinical judgment; a split of 70/30 was performed on the data set followed by feature engineering: range of motion (ROM) was computed through temporal structural network modeling and latent growth curves, whereas pain and fatigue experienced during exercises (reported at the end of each exercise session), exercise accuracy, and time between sessions were computed using growth mixture modeling to depict changes over time. Model development consisted of a tree-based binary classifier and a recurrent neural network that were optimized using the receiver operating characteristic (ROC)-area under the curve (AUC) as the target metric. Precision-recall curves were used as evaluation metrics, and model explainability was assessed using SHAP (Shapley Additive Explanations) values. FN: false negative; FP: false positive; LightGBM: light gradient boosting machine; TN: true negative; TP: true positive.

ROM data from 4 exercises were encoded into a unified composite value that illustrates the longitudinal progression of trunk motion [34], utilizing both latent growth curve analysis (LGCA) [35,36] and temporal structural network modeling (Figure 2) [37]. While the former captures ROM patterns over time to estimate individual trajectories among the 2 classes (pain nonresponders and pain responders), the latter illustrates how improvements in one exercise may be correlated with improvements in other exercises. Among a diverse array of prescribed exercises (N=117), only those that incorporated trunk motion—extension, flexion, rotation, and side bending—were included [34]. This subset was chosen due to its frequent prescription among patients and its superior performance in model comparisons, as determined by likelihood-ratio tests.

Thus, we first constructed the LGCA and temporal structural network model for both responders and nonresponders (*P*<.001) to estimate an intercept, slope, curve, and latent ROM, as well as individual covariance matrices for each patient (see Equations S1-S3 in Multimedia Appendix 1). The proximity of an individual covariance matrix to the expected covariance matrices from responders and nonresponders was assessed using the chi-square measure, which was then incorporated as a feature.

Mixture clustering was used to create new features, using the probabilities of belonging to different clusters to leverage the underlying characteristics of those clusters in making predictions, following a mixture of experts approach. A growth mixture model [38] was applied to cluster longitudinal trajectories of pain and fatigue profiles during exercise, as well as the performance of the exercises and the time spent in sessions. The intent was to capture subpopulations with a high probability of sharing similar patterns (eg, high, moderate, slight, or no improvement). The Bayesian information criterion indicated that 5 clusters provided the optimal fit for the data (see Figure S2 in Multimedia Appendix 1). The individual probability of a patient being assigned to each trajectory class was calculated using likelihood ratios (see Equation S4 in Multimedia Appendix 1), yielding 5 probabilities per participant for each variable at each session.

For the early sessions (ie, sessions 1-3), longitudinal models could not be calculated due to the requirement of at least four time points. Therefore, the raw values or their deltas between sessions were calculated for the outcomes mentioned in this section.

#### Labeling

Pain responders (as defined in the “Outcome” subsection) were labeled as 0, while nonresponders were labeled as 1. This labeling serves as a flag to assist PTs during monitoring and clinical decision-making, thereby representing a binary classification scheme (see Figure 2). Depending on the discharge time point (which occurred no later than the 30th treatment session), the corresponding latest survey (9th, 18th, or 27th) was used to create the labels.

#### Model Pipeline

Two different approaches were used to predict pain response from session 1 to session 7: a tree-based model and a recurrent neural network (RNN) model.

For the tree-based model, a light gradient boosting machine (LightGBM) was utilized, with hyperparameter optimization performed using the Optuna library [39]. This involved 50 trials per algorithm, using the area under the receiver operating characteristic curve (ROC-AUC) as the evaluation metric. LightGBM was trained with 5-fold cross-validation on the training data from each session. As these models are not sequence models, features from previous sessions were concatenated as sessions progressed, allowing models in later sessions to access features from historical sessions. An AUC of 0.5 indicates a random predictor, while an AUC of 1 signifies a perfect predictor. For each fold in the cross-validation, the synthetic minority oversampling technique (SMOTE) was applied to oversample the training data for both classes, reaching 5000 samples per class, as the absence of SMOTE negatively affected model performance. Using the hyperparameters that yielded the best validation performance, a final model was fitted on the entire training set.

An RNN model applied across the 7 sessions can be viewed as a time series (using the PyTorch Python library [40]; Python Foundation). Similar to the tree-based method, the Optuna library was utilized for hyperparameter optimization. However, unlike the tree-based setup, SMOTE was not applied due to its unsuitability for sequential data. RNN models were trained using binary cross-entropy loss and early stopping, with a patience of 10 epochs, utilizing the validation ROC-AUC as the early stopping metric. Using the hyperparameters that yielded the best validation performance, a final model was fitted on the entire training set. This final model was trained with a fixed number of epochs, defined as the average number of early stopping epochs across the cross-validation folds.

The performances of both models were assessed on the test set by calculating the ROC-AUC, precision-recall AUC, *F*_1_-score (at a 0.5 threshold), sensitivity, specificity, and negative predictive value (Figure 2).

#### Model Explainability

Shapley Additive Explanations (SHAP) was applied on the best model to assess model explainability by investigating feature importance [41] (the Python SHAP library [41] for tree-based and the Python timeshare library [42] for RNN models; Figure 2). Positive SHAP values indicate higher prediction scores, meaning a higher probability of nonresponse. To understand the overall impact of different domains, SHAP values for each feature were cumulatively aggregated within their respective domains for each session. As LightGBM models were trained with an increasing number of features over time, SHAP values were normalized per session to examine the relationship between SHAP values and feature values throughout the intervention. Patient SHAP values were visualized using SHAP dependence plots, utilizing a base value of 0.028 and a contribution threshold of 0.05.

Recursive feature elimination, which involved removing features with low SHAP values after hyperparameter optimization, progressively deteriorated model performance.

### Fairness

Demographic and clinical characteristics identified as statistically different between the training and test sets were subjected to subgroup analysis to assess potential bias, including factors such as gender, social deprivation index, age, acuity, and pain levels.

### Model Output

The model output classified patients as responders or nonresponders, based on the previously defined outcome, and was contingent on the applied threshold (eg, 0.95).

### Train and Test Sets

A data split was conducted in which 70% (4313/6125) of the patients were randomly assigned for training the model, while the remaining 30% (1812/6125) were reserved for testing.

### Statistical Analysis

Demographic and clinical presentations at baseline were analyzed using means and proportions. The minimum and maximum ROC-AUC values were calculated by bootstrapping the training and test sets separately (1000 bootstrapped sets). Model comparisons involved ROC-AUCs calculated using the DeLong algorithm [43]. Feature engineering models were developed using R (version 4.2.2; R Foundation for Statistical Computing; packages: lavaan, mlVAR, xgboost, mice, and tidySEM). All other analyses were conducted using Python (version 3.9.7; package: TreeSHAP). The level of significance was set at *P*<.05, considering 2-sided hypothesis tests.

## Results

### Study Population

A total of 6125 patients met the inclusion criteria (see the flowchart of the study cohort in Figure S3 in Multimedia Appendix 1). Among them, 2172 (35.46%) were classified as nonresponders, while 3953 (64.54%) were responders. The analysis encompassed various domains and subdomains, including demographic, socioeconomic, productivity, clinical, prescription, exercise performance, and motivation/compliance data, up to session 7 (see further details in Table 1). Baseline demographic and clinical characteristics for the overall cohort, as well as stratified by training and test data sets, are presented in Table 2. The overall cohort primarily consisted of women (3445/6125, 56.24%), patients aged 41-60 years (3488/6125, 56.95%), fully employed individuals (5119/6125, 83.58%), and college graduates (4037/6125, 65.91%). Additionally, 2335 (38.12%) individuals were classified as obese, 1931 (31.53%) identified as racial or ethnic minorities (ie, Asian, Black, Hispanic, and other), and 1585 (25.88%) came from socially deprived backgrounds.

**Table 2:** Baseline demographics and clinical data.

Demographics		Overall cohort (n=6125)	Train data set (n=4313)	Test data set (n=1812)	*P* value
Age (years), mean (SD)		48.5 (11.3)	48.6 (11.4)	48.2 (10.9)	.25
**Age category (years), n (%)**					.02
	<25	51 (0.83)	42 (0.97)	9 (0.50)	
	25-40	1559 (25.45)	1078 (24.99)	481 (26.55)	
	41-60	3488 (56.95)	2438 (56.53)	1050 (57.95)	
	>60	1027 (16.77)	755 (17.51)	272 (15.01)	
**Gender, n (%)**					.03
	Women	3445 (56.24)	2378 (55.14)	1067 (58.89)	
	Men	2667 (43.54)	1925 (44.63)	742 (40.95)	
	Nonbinary	9 (0.15)	6 (0.14)	3 (0.17)	
	Prefers not to answer	4 (0.07)	4 (0.09)	0 (0)	
BMI (kg/m^2^), mean (SD)^a^		29.5 (6.9)	29.4 (6.9)	29.7 (6.9)	.24
**BMI category, n (%)^a^**					.39
	Underweight (<18.5 kg/m^2^)	48 (0.78)	38 (0.88)	10 (0.55)	
	Normal (18.5-25 kg/m^2^)	1572 (25.67)	1125 (26.08)	447 (24.67)	
	Overweight (≥25-30 kg/m^2^)	2163 (35.31)	1521 (35.27)	642 (35.43)	
	Obese (>30-40 kg/m^2^)	1847 (30.16)	1278 (29.63)	569 (31.40)	
	Morbidly obese (40 kg/m^2^)	488 (7.97)	346 (8.02)	142 (7.84)	
**Race and ethnicity, n (%)**					.005
	Asian	567 (9.26)	388 (9.0)	179 (9.88)	
	Black	564 (9.21)	395 (9.16)	169 (9.33)	
	Hispanic	653 (10.66)	469 (10.87)	184 (10.15)	
	Non-Hispanic White	3524 (57.53)	2442 (56.62)	1082 (59.71)	
	Other	147 (2.40)	105 (2.43)	42 (2.32)	
	Not available/prefers not to specify	670 (10.94)	514 (11.92)	156 (8.61)	
**Employment status, n (%)**					<.001
	Full-time employed	5119 (83.58)	3641 (84.42)	1478 (81.57)	
	Part-time employed	440 (7.18)	265 (6.14)	175 (9.66)	
	Not employed	482 (7.87)	345 (8.00)	137 (7.56)	
	Not available/prefers not to answer	84 (1.37)	62 (1.44)	22 (1.21)	
**Education level, n (%)**					.13
	Less than a high school diploma	61 (1.0)	48 (1.11)	13 (0.72)	
	High school diploma	512 (8.36)	355 (8.23)	157 (8.66)	
	Some college	1515 (24.73)	1089 (25.25)	426 (23.51)	
	Bachelor’s degree	2328 (38.01)	1650 (38.26)	678 (37.42)	
	Graduate degree	1709 (27.90)	1171 (27.15)	538 (29.69)	
**Social Deprivation Index^b^, n (%)**					.81
	Category 1 (0-20)	1887 (30.81)	1316 (30.51)	571 (31.51)	
	Category 2 (21-40)	1495 (24.41)	1052 (24.39)	443 (24.45)	
	Category 3 (41-60)	1136 (18.55)	796 (18.46)	340 (18.76)	
	Category 4 (61-80)	961 (15.69)	686 (15.91)	275 (15.18)	
	Category 5 (81-100)	624 (10.19)	449 (10.41)	175 (9.66)	
**Clinical presentation, mean (SD)**					
	Pain	5.7 (1.4)	5.7 (1.4)	5.7 (1.4)	.36
**Acuity, n (%)**					.24
	Acute	1168 (19.07)	839 (19.45)	329 (18.16)	
	Chronic	4957 (80.93)	3474 (80.55)	1483 (81.84)	
Oswestry Disability Index, mean (SD)		23.1 (12.7)	22.6 (12.5)	24.4 (13.2)	<.001
Fear-Avoidance Beliefs Questionnaire ≥15 (n=1391), mean (SD)		2.8 (18.1)	2.8 (18.1)	2.8 (18.0)	.52
Anxiety (7-item General Anxiety Disorder Scale) ≥5 (n=2144), mean (SD)		3.8 (8.7)	3.9 (8.6)	3.8 (8.7)	.66
Depression (9-item Patient Health Questionnaire) ≥5 (n=1590), mean (SD)		4.2 (9.4)	4.2 (9.4)	4.2 (9.4)	.87
Overall work (Work Productivity and Activity Impairment Questionnaire for General Health overall) score >0 (n=3296), mean (SD)		22.9 (34.0)	22.8 (34.0)	23.0 (33.9)	.85
Activities (Work Productivity and Activity Impairment Questionnaire for General Health) >0 (n=4900), mean (SD)		22.5 (38.8)	22.5 (39.0)	22.4 (38.3)	.32

^a^Denotes 7 missing values.

^b^Denotes 22 missing values (higher quantiles indicate higher social deprivation).

Overall, the cohort reported moderate pain, with an NPRS score of 5.7 out of 10.0 (SD 1.4), and a disability score on the Oswestry Disability Index (ODI) [44] of 23.1 (SD 12.7) at baseline. This was primarily associated with chronic pain, defined as pain lasting 3 months or longer (4957/6125, 80.93%). Severe pain (NPRS: 8-10) was predominantly reported by patients with acute LBP (168/1168, 14.38% vs 519/4957, 10.47%, in chronic cases, *P*<.001). High fear-avoidance beliefs (Fear-Avoidance Beliefs Questionnaire score ≥15 [45]) were reported by 1391 of the 6125 (22.71%) patients of the cohort, while 2144 (35.0%) and 1590 (25.96%) patients reported at least mild anxiety (7-item General Anxiety Disorder Scale [GAD-7] score ≥5 [31]) or depression (9-item Patient Health Questionnaire [PHQ-9] score ≥5 [32]), respectively. A large majority (4900/6125, 80.0%) reported impairment in daily activities (Work Productivity and Activity Impairment Questionnaire for General Health [WPAI]—activity score >0), while 3296 of the 6125 (53.81%) patients reported either presenteeism or absenteeism (WPAI overall score >0). Clinical outcomes were similar across both the training and test data sets, with the exception of the ODI, which was statistically higher in the test data set (22.6, SD 12.5 vs 24.4, SD 13.2), although this difference was not clinically meaningful [46].

### Model Development

The LGCA revealed a significantly different latent representation of trunk motion over time for responders and nonresponders (*P*<.001; Figure S4 in Multimedia Appendix 1). Similarly, the temporal structural network model showed 2 significantly different temporal correlation patterns of trunk motion for responders and nonresponders (*P*<.001; Figure S5 in Multimedia Appendix 1).

**Figure 3:**
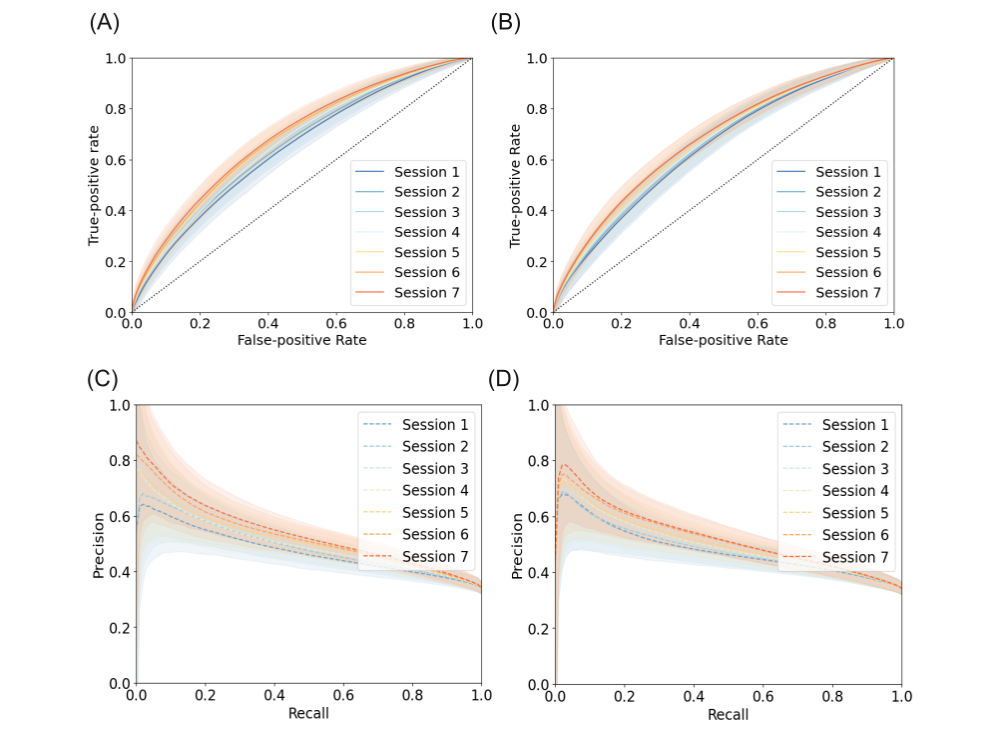
Model performance on the test data set. Test receiver operating characteristic curve (ROC) for both (A) light gradient boosting machine (LightGBM) and (B) recurrent neural network (RNN) across the 7 sessions. True positive rate (true positives over true positives and false negatives); false positive rate (false positives over false positives and true negatives); dashed line denotes an area under the curve (AUC) of 0.5 corresponding to a random predictor; precision-recall curve for (C) LightGBM and (D) RNN models across the 7 sessions. Precision denotes true positives over true positives and false positives; recall denotes true positives over true positives and false negatives; shaded areas denote the 95% CIs.

The best models from both methodologies (LightGBM and RNN), following hyperparameter optimization, resulted in similar ROC-AUC scores in the test set across all time points (*P*=.98, .52, .69, .81, .38, .68, and .22, respectively, for sessions 1-7; Figure 3A and B). Model performance improved over time for both models (*P*<.001 for both, DeLong algorithm), with predictions at session 7 reaching an ROC-AUC of 0.70 (95% CI 0.65-0.71, based on bootstrap resampling; Table S3 in Multimedia Appendix 1) for RNN and 0.71 (95% CI 0.67-0.72) for LightGBM, compared with 0.66 (95% CI 0.61-0.68) and 0.67 (95% CI 0.61-0.67) obtained in session 1. These results corresponded to a precision-recall AUC at session 7 of 0.56 (LightGBM: 95% CI 0.49-0.58; RNN 95% CI 0.48-0.57; Figure 3C and D) and a weighted *F*_1_-score of 0.68 (95% CI 0.64-0.69) for both models.

For the models described, considering a precision of 70%, the LightGBM achieved a specificity of 0.97 (95% CI 0.95-0.98), a sensitivity of 0.13 (95% CI 0.02-0.25), and a negative predictive value of 0.68 (95% CI 0.68-0.68). Similarly, the RNN achieved a specificity of 0.97 (95% CI 0.94-0.97), a sensitivity of 0.12 (95% CI 0.00-0.24), and a negative predictive value of 0.68 (95% CI 0.65-0.71).

### Predictive Factors in Digital Care

**Figure 4:**
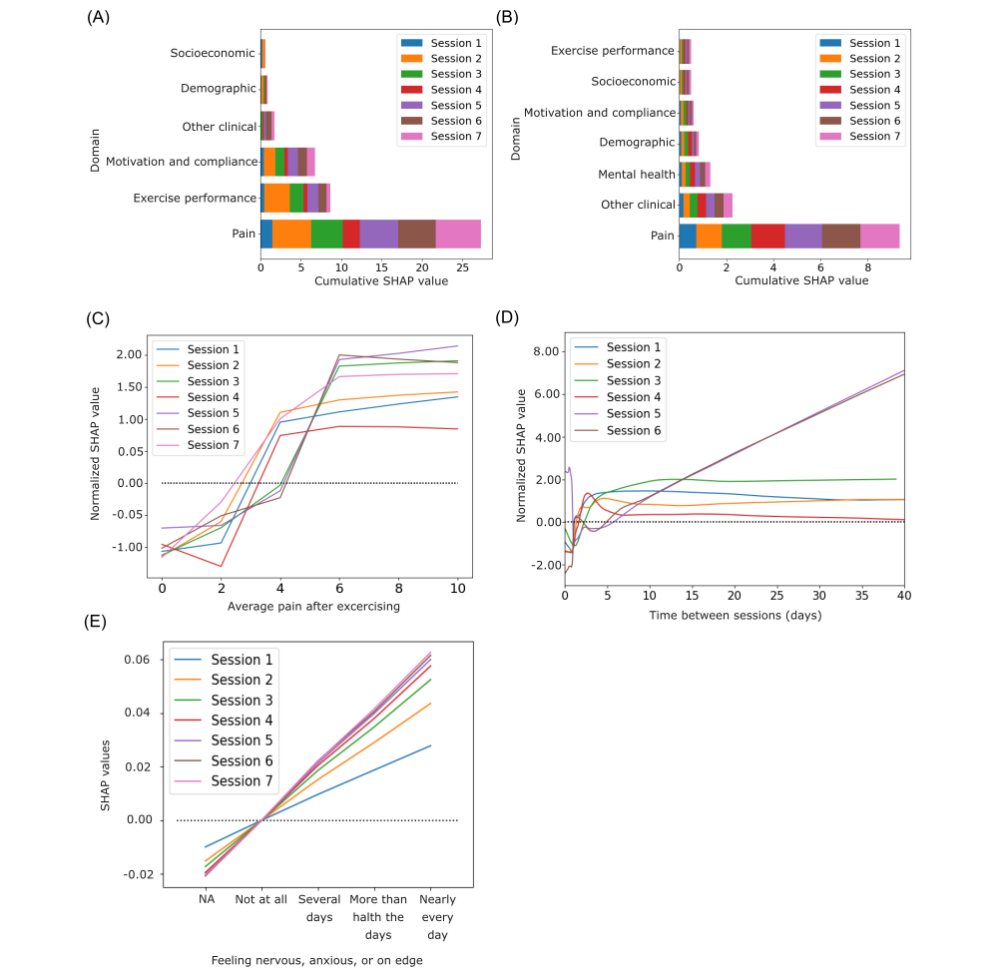
Model explainability. Cumulative Shapley Additive Explanations (SHAP) values per domain considering the top 20 features at each session for both (A) light gradient boosting machine (LightGBM) and (B) recurrent neural network (RNN). SHAP values depicting the relationship between the outcome (ie, pain response) and the feature of interest: (C) average pain felt during exercising, (D) time between sessions (days), and (E) feeling nervous, anxious, or on edge (7-item General Anxiety Disorder Scale [GAD-7] scale). As LightGBM models differ in the number of features across time, and because we are interested in qualitative comparisons, SHAP values were normalized at each session by dividing by the SD of SHAP values.

Considering the best models, we analyzed the top 20 outcome predictors at each session based on absolute mean SHAP values [41]. Figure 4 depicts cumulative values of features aggregated by domains over time for the LightGBM (Figure 4A) and RNN (Figure 4B). Detailed feature stratification for both models is available in Figures S6 and S7 in Multimedia Appendix 1, respectively.

Although both models incorporated features from comparable domains, the importance of each feature varied depending on the model. The flagged domains included pain-associated metrics, exercise performance, and motivation and compliance data, which utilized real-time passive data collection.

Although the pain domain was highly predictive for both models (Figures 4A and B), LightGBM placed greater emphasis on exercise performance, motivation, and compliance data than the RNN. Within the exercise performance domain, the most informative features included time spent exercising, exercise accuracy, and the execution of specific exercises related to trunk motion. Notably, lower training time or poorer performance in particular sessions was associated with an increased likelihood of nonresponse (Figure S8A in Multimedia Appendix 1). Low motivation and compliance, specifically characterized by low exercise consistency—evaluated by longer intervals between sessions (>3 days, Figure 4D)—was associated with a higher likelihood of being a nonresponder. This observation was consistent across other variables reflecting motivation toward compliance, such as the time between registration and program start, as well as the reasons preventing patients from completing the program.

The RNN was more reliant on the temporal patterns of mental health and other clinical variables (eg, medication intake, intent to undergo surgery) for its predictions. The presence of mental distress was associated with a higher likelihood of being a nonresponder across all sessions, as exemplified by the item “Feeling nervous, anxious, or on edge” from the GAD-7 scale (Figure 4E). Even though LightGBM did not rank mental health features among the top 20 predictors, higher GAD-7 and PHQ-9 scores were correlated with poorer outcomes, particularly in the later sessions (eg, Figure S8B in Multimedia Appendix 1). Additionally, other clinical features, such as prescribed medication, were associated with a lower probability of being a responder.

Both models utilized features related to individual characteristics, including demographics and socioeconomic status. Subgroup analyses evaluating potential imbalances between the training and test sets indicated that similar results could be obtained regardless of demographic or clinical characteristics (Table S4 in Multimedia Appendix 1). Overall, women and individuals from more socially deprived areas, particularly those from regions with higher unemployment rates, had a worse prognosis compared with their counterparts (Figure S8C and S8D in Multimedia Appendix 1).

### Model Applicability

**Figure 5:**
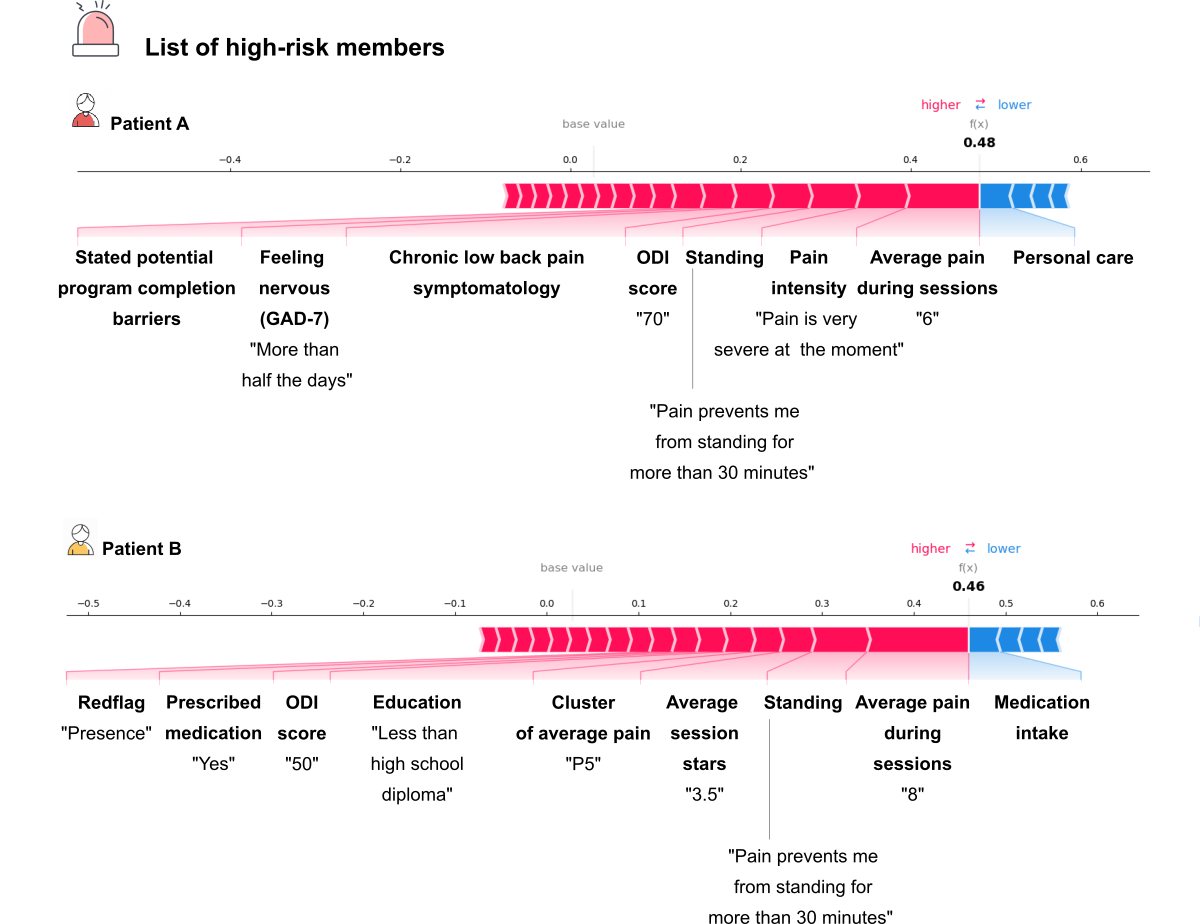
Example of the integration of the model in the physical therapist portal. Example of model explainability for 2 patients, A and B, classified as high-risk nonresponders (recurrent neural network [RNN] precision 70%) at session 4. In the physical therapist portal, each patient's health record includes the prediction of the tool along with the variables that sustained the model's classification, providing insights into the specific factors affecting the patient's prognosis. The most influential factors contributing to a nonresponder prediction are highlighted in red, with factors positioned toward higher "base values" (ie, to the right) indicating a stronger impact. For example in the case of patient A, the pain felt during exercise and the baseline pain are the most influential for the model output. After analyzing the case, the physical therapist reevaluates the patient's program and the potential need for further support. Those adjustments will be subsequently fed into the tool reinforcing the dynamic optimization close loop. GAD-7: 7-item General Anxiety Disorder Scale; ODI: Oswestry Disability Index.

Figure 5 illustrates an example of the output from the tool provided on the PT portal for 2 patients during session 4. This output includes the model’s prediction of nonresponders (ie, true positives, with an RNN precision of 70% on the test set) and the key drivers behind this classification. For patient A, the classification is primarily influenced by high pain reported during exercise in session 4 (NPRS = 6 out of 10), baseline characteristics indicating severe clinical presentation (such as high ODI and mental distress), and self-reported potential barriers to motivation. Patient B also reported high pain during exercise in session 4 (NPRS = 8 out of 10) and exhibited low exercise performance for that session (3.5 out of 5 stars). Additionally, patient B indicated prescribed medication intake (eg, opioids) and the presence of at least one clinical red flag, despite being cleared by a physician to participate in the program. With an ODI score of 50 and low educational attainment, this information is subsequently analyzed by the PT, allowing for necessary refinements to the intervention. This may involve adjusting exercise intensity or type or prioritizing specific components of the DCP, such as education and cognitive behavioral therapy. These adjustments will subsequently impact future model predictions in a dynamic closed-loop optimization that contributes to the refinement of the tool.

## Discussion

### Principal Findings

This study leveraged passively and automatically collected patient data from a digital MSK intervention to develop a predictive tool that assists PTs in optimizing treatment. These models utilize continuously updated data from each enrolled patient to identify early those who are less likely to achieve a clinically meaningful reduction in pain. Model performance significantly increased over time, achieving an ROC-AUC of 0.70 (95% CI 0.65-0.71, from bootstrap resampling) for the RNN and 0.71 (95% CI 0.67-0.72) for the LightGBM at session 7. These models identified features from domains related to pain, exercise performance, motivation, and compliance as the most predictive of outcomes. This information can inform PTs for continuous treatment adjustments to maximize response rates. This predictive tool operates through a dynamic loop, and when applied in clinical practice, it continuously enhances treatment refinement and improves tool performance over time.

### Comparison Prior Work

Previous ML models designed to support LBP management without real-time data collection have reported performances (ROC-AUCs) ranging from 0.49 to 0.71 [6,7,9,10,21], with 1 model achieving a higher AUC of 0.84, but only near the end of treatment [11]. Additionally, the currently used STarT Back prediction tool has reported AUCs of 0.63 and 0.60-0.62 when predicting pain intensity at 12 weeks and 1 year, respectively [6,7]. The wide range of reported model performances reflects not only the challenges associated with each proposed objective but also the level of uncertainty that these tools generate. In this study, both models’ performances significantly improved over time, achieving an AUC of 0.70-0.71 at session 7 using real-world data. This AUC suggests that pain response at an early stage of the intervention is partially explained by the variables used. However, as treatment progresses, it is plausible that model performance will continue to improve toward the end. As an example, Brennan et al [11] demonstrated a 1.3 times increase in model performance when using PROMs collected at session 3 to predict outcomes at session 6. Our study aimed to be less reliant on PROMs and more focused on a scalable solution capable of improving over time [47,48]. While specificity and sensitivity depend on the threshold applied, the results presented here align with those previously reported [6,7,9-11,21]. The choice of threshold to balance precision and recall must align with the clinical setting’s needs, taking into account the costs and implications of false positives and false negatives.

In addition to real-time predictions, these models may offer timely insights that enable PTs to determine the need for referring patients to specialized care (eg, escalation to psychologists) through shared decision-making with the patient. Both models identified similar feature domains, including pain-associated metrics, exercise performance, motivation, and compliance, thereby surpassing the predictive value of individual demographic prognostic factors. These domains cannot be captured solely through PROMs, further emphasizing the relevance of such models in managing LBP.

To predict the pain that patients experience during daily activities, the model incorporates pain levels reported during exercise, alongside baseline clinical presentations (including the NPRS, the “pain intensity” item from the ODI, and acuity)—features previously established as poor prognostic indicators [49-51]. This underscores the importance of pain experienced during exercise as a predictor of outcomes. Specifically, exercising with pain levels exceeding 4 out of 10 has been shown to predict negative outcomes [52], suggesting that these patients may benefit from adjustments to their exercise prescriptions. The use of analgesic medications, such as opioids, has also been linked to a poorer prognosis, as noted in previous studies [53-55].

Aside from pain, both models prioritized motivation, compliance, and exercise performance data for predictions. Regarding motivation, indicators such as self-reported resistance to commit to the intervention, delayed program initiation, and extended intervals between sessions (>3 days) were associated with a lower likelihood of response, reinforcing previous findings [56]. In the exercise performance domain, the models emphasized exercise execution data, including the time dedicated to sessions, performance scores, and the number of incorrect exercises, with lower scores linked to worse outcomes. Challenges associated with compliance frequently contribute to increased frustration and decreased adherence to treatment [57,58] and have been previously described as risk factors for poor outcomes [5,59]. Co-occurring with low adherence rates, anxiety and depression were associated with a lower likelihood of response in one of the models (RNN). This finding is unsurprising, considering the well-established negative feedback loop between mental health and MSK pain [60,61].

Although the demographic and socioeconomic profiles of patients are relevant, they may be of lower importance than the previously mentioned domains. Certain groups, such as women and the unemployed, may encounter systemic barriers that hinder their ability to achieve pain relief.

In summary, implementing this tool would enhance care coordination and patient management by identifying individuals at higher risk for poor pain outcomes. This, in turn, can streamline workflows, fostering informed decision-making and tailored treatment programs. The continuous stream of data generated by patients enables refined predictions at each interaction, allowing PTs to make timely adjustments to the program. These adjustments could then be fed back into the system to further enhance its recommendations. To assess potential biases, the subgroup analyses conducted in this study focused on demographic characteristics and socioeconomic factors, indicating a low risk of bias. However, further evaluation of algorithmic bias will be necessary once the model is fully operational and deployed in real-world settings. Continuous monitoring will be essential to ensure that the model does not disproportionately impact specific populations or reinforce existing health disparities.

While there may be concerns among PTs regarding the “black box” nature of AI, model explainability will be crucial in overcoming this barrier. Before full implementation, training for PTs (or clinicians) is essential to ensure they understand and can effectively use the tool, thereby mitigating fears and preventing erroneous use of AI. Nevertheless, the design of this AI tool ensures that final clinical decisions remain with the PT, preserving human judgment and critical reasoning. This approach can foster trust in the system and promote the safe integration of AI into clinical workflows.

Future research on the development of this predictive tool should concentrate on clinically validating the closed feedback loop. Additionally, optimizing the performance of the AI tool may be further enhanced by incorporating supplementary data sources, such as real-life context data variables obtainable from smartwatches (eg, pedometry and sleep quality) and clinical context information (eg, presence of specific comorbidities and clinical records). This could reveal additional data patterns and, consequently, contribute to improved model accuracy. With the nearly exponential increase in data, it is plausible that RNNs or other deep sequence models will continue to enhance their performance and eventually surpass LightGBM, given their natural ability to handle sequential data [47,48]. Additionally, the integration of continuous volumes of data may expand the model’s predictive capabilities to assess long-term outcomes. In particular, for LBP, predicting long-term outcomes will help determine which types of interventions lead to more sustained recovery with functional improvement, and reduce the likelihood of relapse or chronicity over time.

Finally, as the AI model relies on protected health information, implementing robust safeguards and establishing data stewardship protocols are essential to maintain confidentiality, integrity, and security.

Other future considerations include exploring the application of such tools to other MSK conditions.

### Strengths and Limitations

This study has important strengths. First is the novelty of exploring how data collected from a DCP can be applied in an ML model to continuously predict patient outcomes and foster personalized care. Second, this cohort’s demographic and clinical characteristics mirror that of the US population reporting LBP [62,63], with the percentage of nonresponders for pain being consistent with previous studies evaluating in-person and digital LBP management [11,22,64,65]. Finally, the nature of the data that are automatically and passively collected, which are less reliant on PROMs, makes it easy to obtain and less burdensome for both patients and clinicians. However, this study also has limitations that warrant noting: (1) Although the cohort was large and decentralized, we cannot dismiss the possibility of overfitting; additionally, as the population focuses on beneficiaries of health plans, this may limit generalizability to populations not covered by health insurance. Therefore, extended testing on new cohorts is critical for the external validation of the AI tool. Such validation would provide a more accurate assessment of the model’s performance, ensuring that its effectiveness and robustness are maintained across different populations that may enroll in the program. Additionally, it could help identify areas in need of improvement. Furthermore, external validation studies should be followed by real-world applications to evaluate the usefulness of the deployed model in clinical settings; (2) despite being highly used and recommended by clinical guidelines, transforming the pain outcome into a binary classification may obscure subtle variations in patient responses. This simplification could lead to misclassification of patients who are close to the threshold. Future approaches could consider modeling the outcome as a continuous variable. Additionally, developing a composite score that reflects improvements across multiple core outcome domains—including Patient Global Impression of Change and Activities of daily living functionality—could better capture the multifactorial nature of LBP and provide a more comprehensive assessment of patient improvement in a single metric; (3) we used an extensive number of features that capture critical domains in the rehabilitation realm; however, other potential features, such as measures of self-efficacy and indicators depicting transitional states between clusters of trajectories, which could hold high predictive power, were not included. Incorporating these features in future designs could enhance the model’s predictive accuracy; (4) finally, the early stage of this tool’s development hampers the ability to assess its impact on clinical outcomes. Future steps should focus on implementing and evaluating the tool’s dynamic loop to better understand its effectiveness and feasibility, as well as to ascertain its net benefits.

### Conclusions

This study underscores the potential of a predictive tool leveraging ML models to enhance the management of LBP in a digital setting, redirecting care pathways early on and throughout the treatment course, thereby fostering personalized care. The application of such a strategy is particularly important for managing a heterogeneous population with diverse phenotypes across different cultural contexts.

## Appendix

Multimedia Appendix 1 TRIPOD-AI guidelines and additional analysis and results. TRIPOD-AI: Transparent Reporting of a Multivariable Prediction Model for Individual Prognosis or Diagnosis—artificial intelligence.

## Abbreviations

AI: artificial intelligence
AUC: area under the curve
DPC: digital care program
GAD-7: 7-item General Anxiety Disorder Scale
IMMPACT: Initiative on Methods, Measurement, and Pain Assessment in Clinical Trials
LBP: low back pain
LGCA: latent growth curve analysis
LightGBM: light gradient boosting machine
ML: machine learning
MSK: musculoskeletal
NPRS: Numerical Pain Rating Scale
ODI: Oswestry Disability Index
PROM: patient-reported outcome measure
PT: physical therapist
RNN: recurrent neural network
ROC: receiver operating characteristic
ROM: range of motion
SHAP: Shapley Additive Explanations
SMOTE: synthetic minority oversampling technique
TRIPOD-AI: Transparent Reporting of a Multivariable Prediction Model for Individual Prognosis or Diagnosis—artificial intelligence
WPAI: Work Productivity and Activity Impairment Questionnaire for General Health

## Data Availability

The data sets used or analyzed during this study are available from the corresponding author upon reasonable request.

## Code Availability

The underlying code for this study and the training/validation data sets are not publicly available but may be made accessible to qualified researchers upon reasonable request from the corresponding author.
