# Supplementary material for: Predicting Pain Response to a Remote Musculoskeletal Care Program for Low Back Pain Management: Development of a Prediction Tool
Source: JMIR Med Inform. 2024 Nov 19;12:e64806. doi: 10.2196/64806 (PMC11615557; doi:10.2196/64806)
Supplement: Multimedia Appendix 1 [file medinform_v12i1e64806_app1.pdf]

Supplementary Material

Supplementary Table 1 - Tripod-AI guidelines.

Section/Topic	ı	Checklist Item	Page
**Title and abstract**			
Title	1	Identify the study as developing or evaluating the performance of a multivariable prediction model, the target population, and the outcome to be predicted	1
**Abstract**			
Abstract	2	See TRIPOD+AI for Abstracts checklist	1
**Introduction**			
Background	3a	Explain the healthcare context (including whether diagnostic or prognostic) and rationale for developing or evaluating the prediction model, including references to existing models	2
	3b	Describe the target population and the intended purpose of the prediction model in the context of the care pathway, including its intended users (e.g., healthcare professionals, patients, public)	2
	3c	Describe any known health inequalities between sociodemographic groups	2
	3d	Specify the study objectives, including whether the study describes the development or validation of a prediction model (or both)	2/3
Objectives	4	Specify the study objectives, including whether the study describes the development or validation of a prediction model (or both)	2/3
**Methods**			
Data	4a	Describe the sources of data separately for the development and evaluation datasets (e.g., randomised trial, cohort, routine care or registry data), the rationale for using these data, and representativeness of the data.	4
	4b	Specify the dates of the collected participant data, including start and end of participant accrual; and, if applicable, end of follow-up	3
Participants	6a	Specify key elements of the study setting (e.g., primary care, secondary care, general population) including the number and location of centres	3
	6b	Describe the eligibility criteria for study participants	3
	6c	Give details of any treatments received, and how they were handled during model development or evaluation, if relevant	3
Data preparation	7	Describe any data pre-processing and quality checking, including whether this was similar across relevant sociodemographic groups	4
Outcome	8a	Clearly define the outcome that is being predicted and the time horizon, including how and when assessed, the rationale for choosing this outcome, and whether the method of outcome assessment is consistent across sociodemographic groups	4
	8b	If outcome assessment requires subjective interpretation, describe the qualifications and demographic characteristics of the outcome assessors	NA
	8c	Report any actions to blind assessment of the outcome to be pre	NA
Predictors	9a	Describe the choice of initial predictors (e.g., literature, previous models, all available predictors) and any pre-selection of predictors before model building	4
	9b	Clearly define all predictors, including how and when they were measured (and any actions to blind assessment of predictors for the outcome and other predictors)	4
	9c	If predictor measurement requires subjective interpretation, describe the qualifications and demographic characteristics of the predictor assessors	4, table 1
Sample size	10	Explain how the study size was arrived at (separately for development and evaluation), and justify that the study size was sufficient to answer the research question. Include details of any sample size calculation	7
Missing data	11	Describe how missing data were handled. Provide reasons for omitting any data	8
Analytical methods	12a	Describe how the data were used (e.g., for development and evaluation of model performance) in the analysis, including whether the data were partitioned, considering any sample size requirements	8,9
	12b	Depending on the type of model, describe how predictors were handled in the analyses (functional form, rescaling, transformation, or any standardisation).	8,9
	12c	Specify the type of model, rationale2, all model-building steps, including any hyperparameter tuning, and method for internal validation	9

	12d	Describe if and how any heterogeneity in estimates of model parameter values and model performance was handled and quantified across clusters (e.g., hospitals, countries). See TRIPOD-Cluster for additional considerations3	9
	12e	Specify all measures and plots used (and their rationale) to evaluate model performance (e.g., discrimination, calibration, clinical utility) and, if relevant, to compare multiple models	9,10
	12f	Describe any model updating (e.g., recalibration) arising from the model evaluation, either overall or for particular sociodemographic groups or settings	10
	12g	For model evaluation, describe how the model predictions were calculated (e.g., formula, code, object, application programming interface)	9
Class imbalance	13	If class imbalance methods were used, state why and how this was done, and any subsequent methods to recalibrate the model or the model predictions	9
Fairness	14	Describe any approaches that were used to address model fairness and their rationale	10
Model output	15	Specify the output of the prediction model (e.g., probabilities, classification). Provide details and rationale for any classification and how the thresholds were identified	10
Training versus evaluation	16	Identify any differences between the development and evaluation data in healthcare setting, eligibility criteria, outcome, and predictors	10
Ethical approval	17	Name the institutional research board or ethics committee that approved the study and describe the participant-informed consent or the ethics committee waiver of informed consent	3
**Open Science**			
Funding	18a	Give the source of funding and the role of the funders for the present study	19
Conflicts of interest	18b	Declare any conflicts of interest and financial disclosures for all authors	19
Protocol	18c	Indicate where the study protocol can be accessed or state that a protocol was not prepared	NA
Registration	18d	Provide registration information for the study, including register name and registration number, or state that the study was not registered	3
Data sharing	18e	Provide details of the availability of the study data	18
Code sharing	18f	Provide details of the availability of the analytical code4	18
**Patient & Public Involvement**			
Patient & Public Involvement	19	Provide details of any patient and public involvement during the design, conduct, reporting, interpretation, or dissemination of the study or state no involvement.	NA
**Results**			
Participants	20a	Describe the flow of participants through the study, including the number of participants with and without the outcome and, if applicable, a summary of the follow-up time. A diagram may be helpful.	10, 11, supplementary materials
	20b	Report the characteristics overall and, where applicable, for each data source or setting, including the key dates, key predictors (including demographics), treatments received, sample size, number of outcome events, follow-up time, and amount of missing data. A table may be helpful. Report any differences across key demographic groups.	10,11, table 2
	20c	For model evaluation, show a comparison with the development data of the distribution of important predictors (demographics, predictors, and outcome).	Supplementary table 3
Model development	22	*Specify the number of participants and outcome events in each analysis (e.g., for model development, hyperparameter tuning, model evaluation)*	10
Model specification	22	Provide details of the full prediction model (e.g., formula, code, object, application programming interface) to allow predictions in new individuals and to enable third-party evaluation and implementation, including any restrictions to access or re-use (e.g., freely available, proprietary)5	13
Model performance	23a	Report model performance estimates with confidence intervals, including for any key subgroups (e.g., sociodemographic). Consider plots to aid presentation.	13
	23b	If examined, report results of any heterogeneity in model performance across clusters. See TRIPOD Cluster for additional details3.	13,14
Model updating	24	Report the results from any model updating, including the updated model and subsequent performance	NA
**Discussion**			

Interpretation	25	Give an overall interpretation of the main results, including issues of fairness in the context of the objectives and previous studies	15
Limitations	18	Discuss any limitations of the study (such as a non-representative sample, sample size, overfitting, missing data) and their effects on any biases, statistical uncertainty, and generalizability	17-18
Usability of the model in the context of current care	19b	Describe how poor quality or unavailable input data (e.g., predictor values) should be assessed and handled when implementing the prediction model	15-17
		Specify whether users will be required to interact in the handling of the input data or use of the model, and what level of expertise is required of users	15-17
	20	Discuss any next steps for future research, with a specific view to applicability and generalizability of the model	15-17

Supplementary Table 2 - Percentage of missing values in the variables within high predictive power.

**Variable**	**Percent of Missing Values (%)**
Pain Baseline	0
Exercise Performance	
Session 1	0.64
Session 2	0.23
Session 3	0.15
Session 4	0.18
Session 5	0.29
Session 6	0.59
Session 7	0.95
Time Spent exercising	
Session 1	0.02
Session 2	0.02
Session 3	0.02
Session 4	0.03
Session 5	0.05
Session 6	0.39
Session 7	0.82
Exercise accuracy	
Session 1	0.02
Session 2	0.02
Session 3	0.02
Session 4	0.03
Session 5	0.05
Session 6	0.39
Session 7	0.82
ROM of trunk rotation	
Session 1	39.40
Session 2	35.09
Session 3	35.85
Session 4	45.34

Session 5	47.33
Session 6	45.08
Session 7	47.33
Time between sessions	
Session 2	0.02
Session 3	0.02
Session 4	0.03
Session 5	0.05
Session 6	0.39
Session 7	0.82
Time between registration and program-start	0
Reasons to hold to complete the program	12.4
Feeling nervous or on in edge	1.1
GAD-7	1.1
PHQ-9	1.1
Age	0
Gender	0
Social Deprivation index	0.36

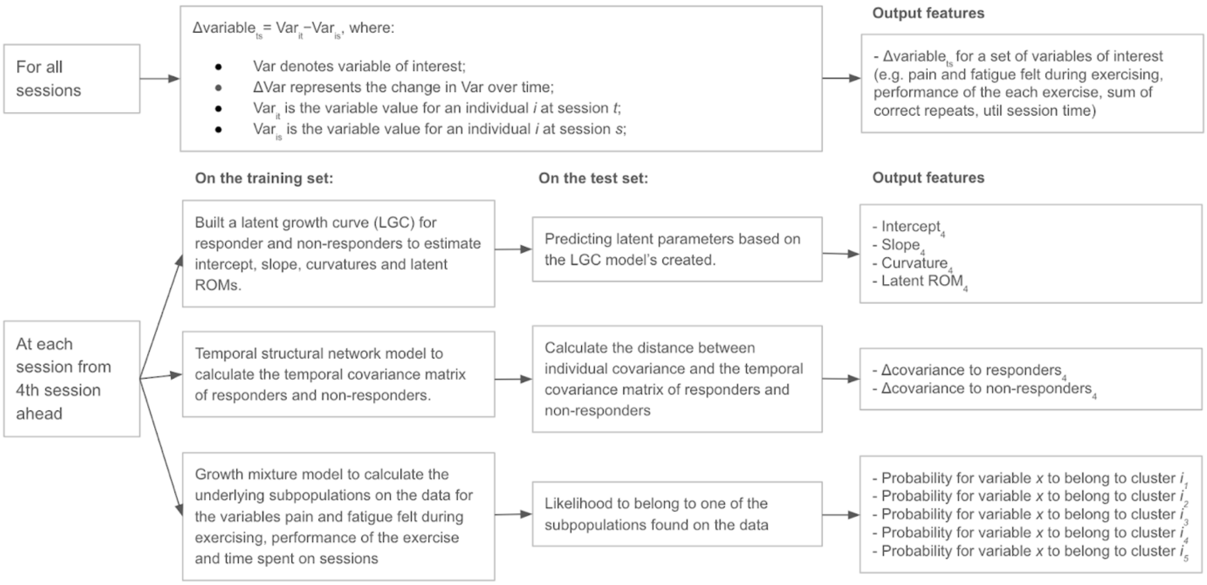

Supplementary Figure 1 - Detailed process used for feature engineering.

Mathematical equations

$$ROM_t^g = I^g + S_t^g + C^g t^2 + e_t^g \text{(Equation 1)}$$

$$rom^g{}_{ti} = \mu_{ti} + \lambda_{ti} ROM^g{}_t + \epsilon^g{}_t \text{ (Equation 2)}$$

Where $ROM^g{}_t$ is a latent ROM variable at each time point; I, S, and C are latent intercept, slope, and curve parameters defining longitudinal trajectories of ROM per class; $rom^g{}_{ti}$ is a measured ROM variable, i, at time, t, for class g; μ_{ti} is a vector of means of each ROM variable at each time point; $\lambda_{ti}$is a vector of factor loadings relating observed ROM scores to latent ROMscores; and $e^g{}_t$ and $\epsilon^g{}_t$represent model error and measurement error respectively.

$$\sum = [(\mathrm{I} - \mathrm{B})\mathrm{T} - 1(\mathrm{I} - \mathrm{B})] - 1 \quad \text{(Equation 3)}$$

The chi-square difference values obtained from the comparison between the individual covariance matrix and the covariance matrix of each class. In this equation is a model expected covariance matrix; I is an identity matrix; B is a parameter matrix; and is a diagonal matrix containing error variance components.

$$x_t = \sum_{g=1}^{G} p_g (I^g + S^g t + C^g t^2) + e_t \text{ (Equation 4)}$$

Where $x_t$is a vector of observations at time t; g is the total number of mixture clusters assessed; I^g, S_g, and C^g are latent intercept slope and curve parameters for each cluster; $p_g$denotes a vector of probabilities of belonging to a cluster; and e_t is an error term.

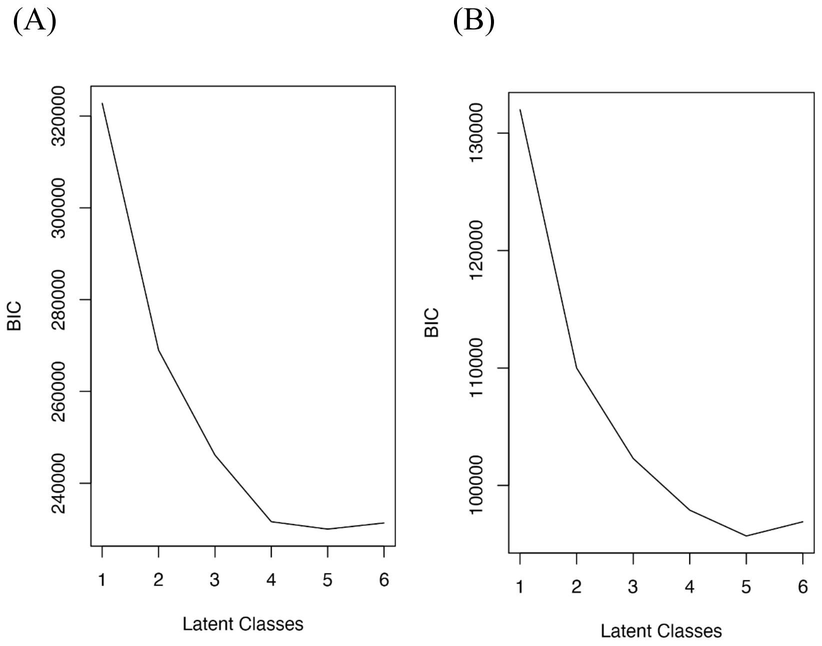

Supplementary Figure 2 - Bayesian information criteria (BIC) for the growth mixture modeling of (A) pain (NPRS) and (B) fatigue.

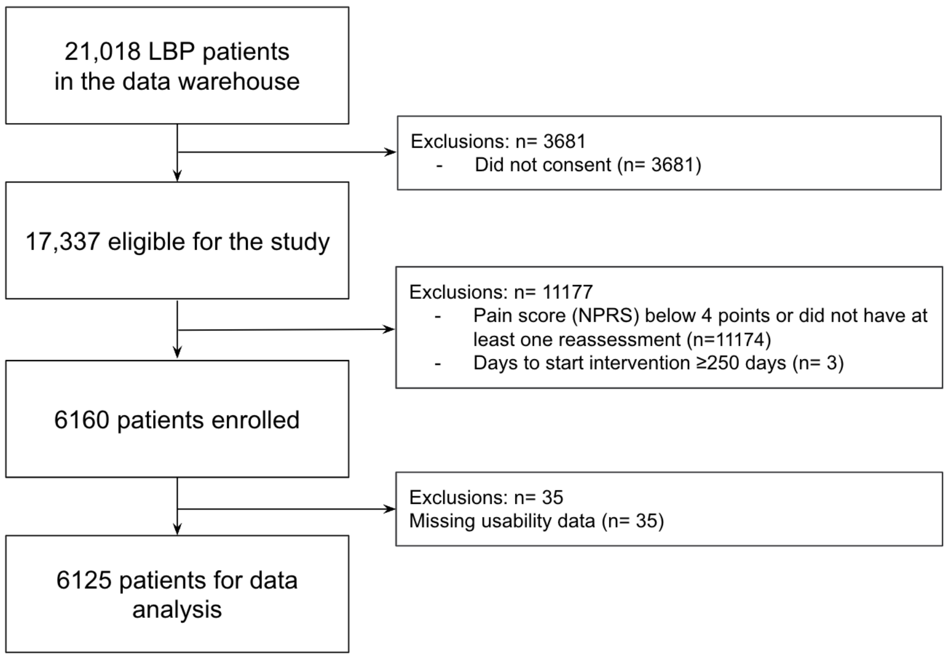

Supplementary Figure 3 - Flow chart of study cohort.

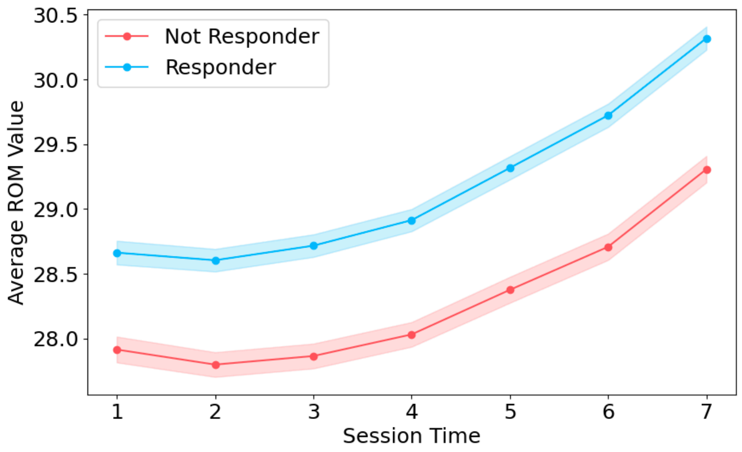

Supplementary Figure 4 - Latent representation of average range of motion over time for pain responders and non-responders. Non-responders: patients who did not experience a pain (NPRS) reduction of 30% nor reported pain level lower than 4 at program-end.

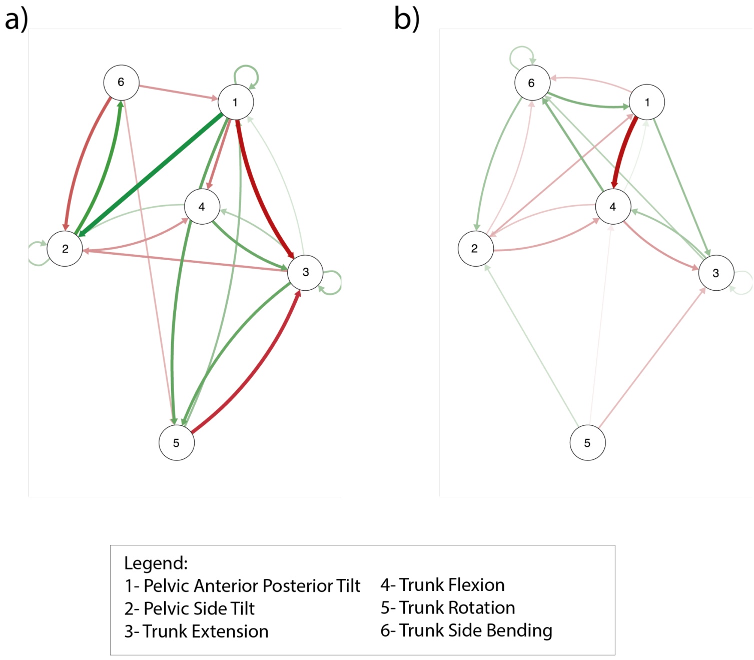

Supplementary Figure 5 - Schematic representation of the structural network model for (a) non-responders and (b) responders. The image corresponds to an illustration that does not depict all elements considered for the analysis. Red lines denote negative temporal correlation, while green denotes positive temporal correlation. Thickness of the edges represents the strength of the correlation.

Supplementary table 3 - Summarized performance metrics of the LightGBM and RNN at 7th session.

Model	AUC		PR-AUC		F1 score		Specificity		Sensitivity		Negative Predictive Value	
	Best model	95%CI	Best model	95%CI	Best model	95%CI	Best model	95%CI	Best model	95%CI	Best model	95%CI
**LightGBM**	0.71	0.67;0.72	0.56	0.49;0.58	0.68	0.64;0.69	0.97	0.95;0.98	0.13	0.02;0.25	0.68	0.68;0.68
**RNN**	0.70	0.65;0.71	0.56	0.48;0.57	0.68	0.64;0.69	0.97	0.94;0.97	0.12	0.00;0.24	0.68	0.65;0.71

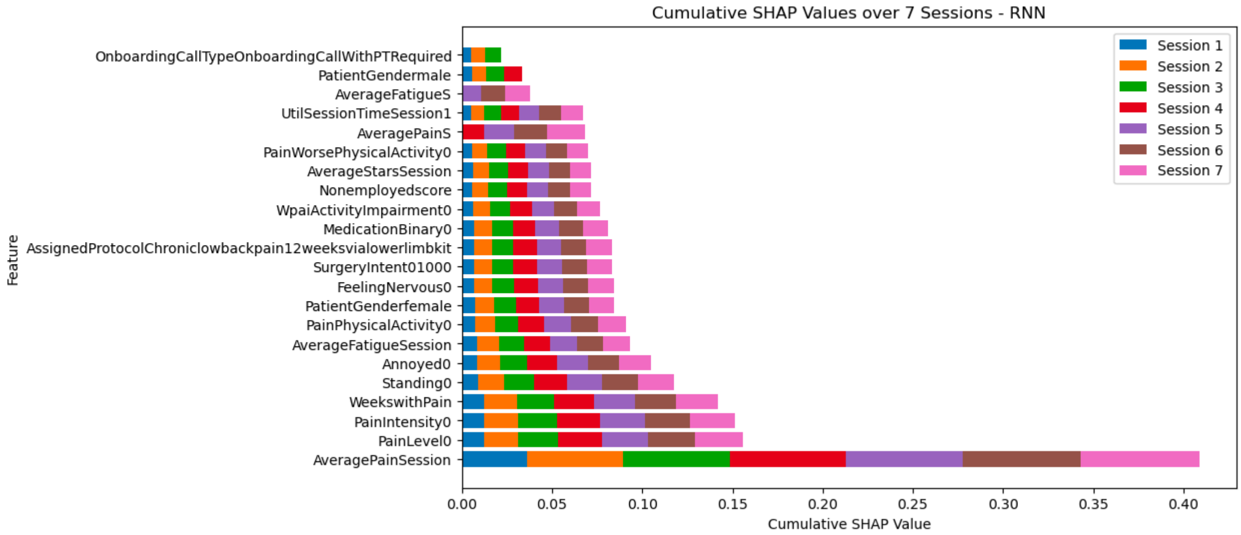

Supplementary Figure 6- SHAP values per feature were computed for each session across variables ranked in the top 20 by the RNN model.

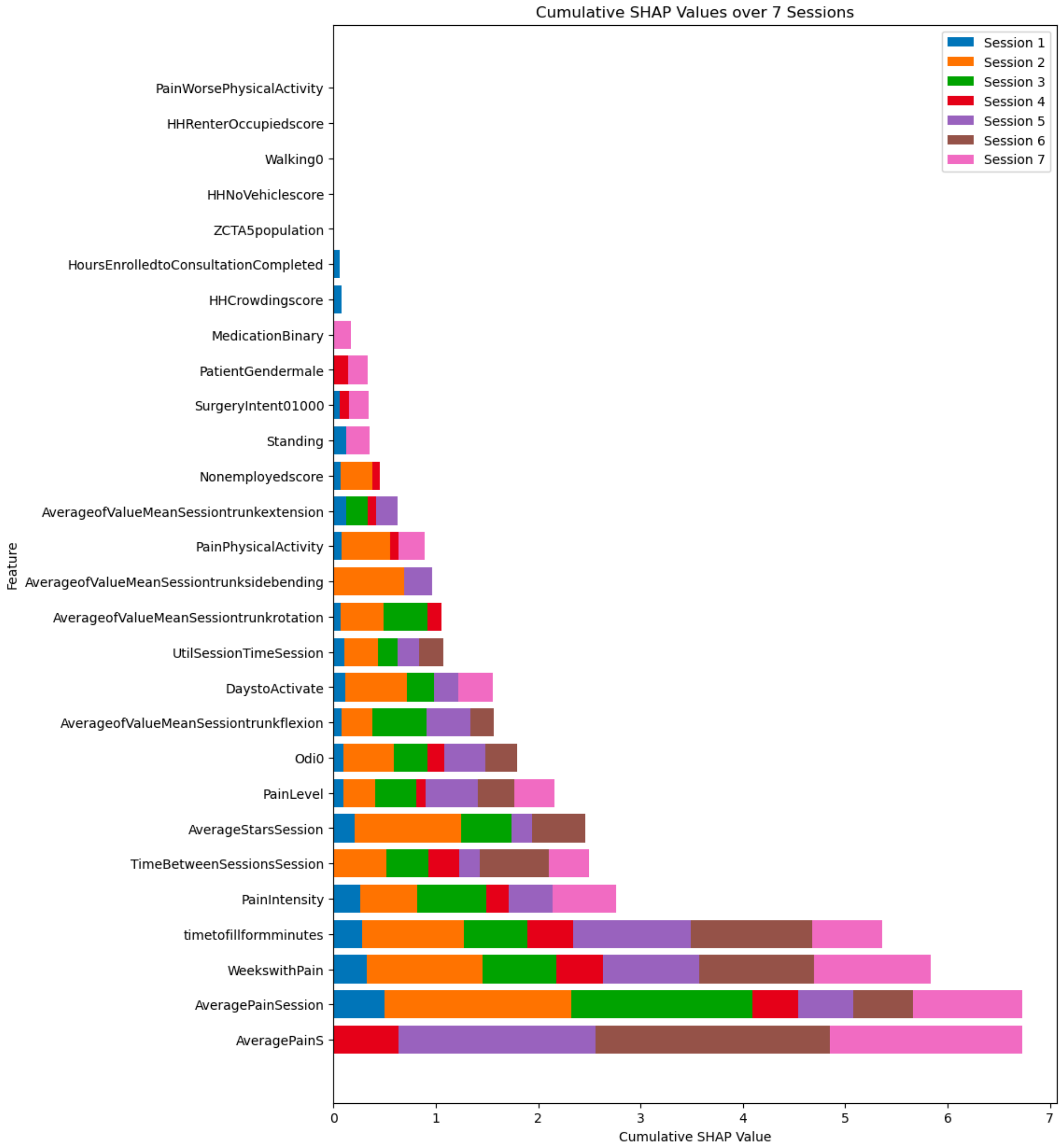

Supplementary Figure 7 - SHAP values per feature were computed for each session across variables ranked in the top 20 by the LightGBM model.

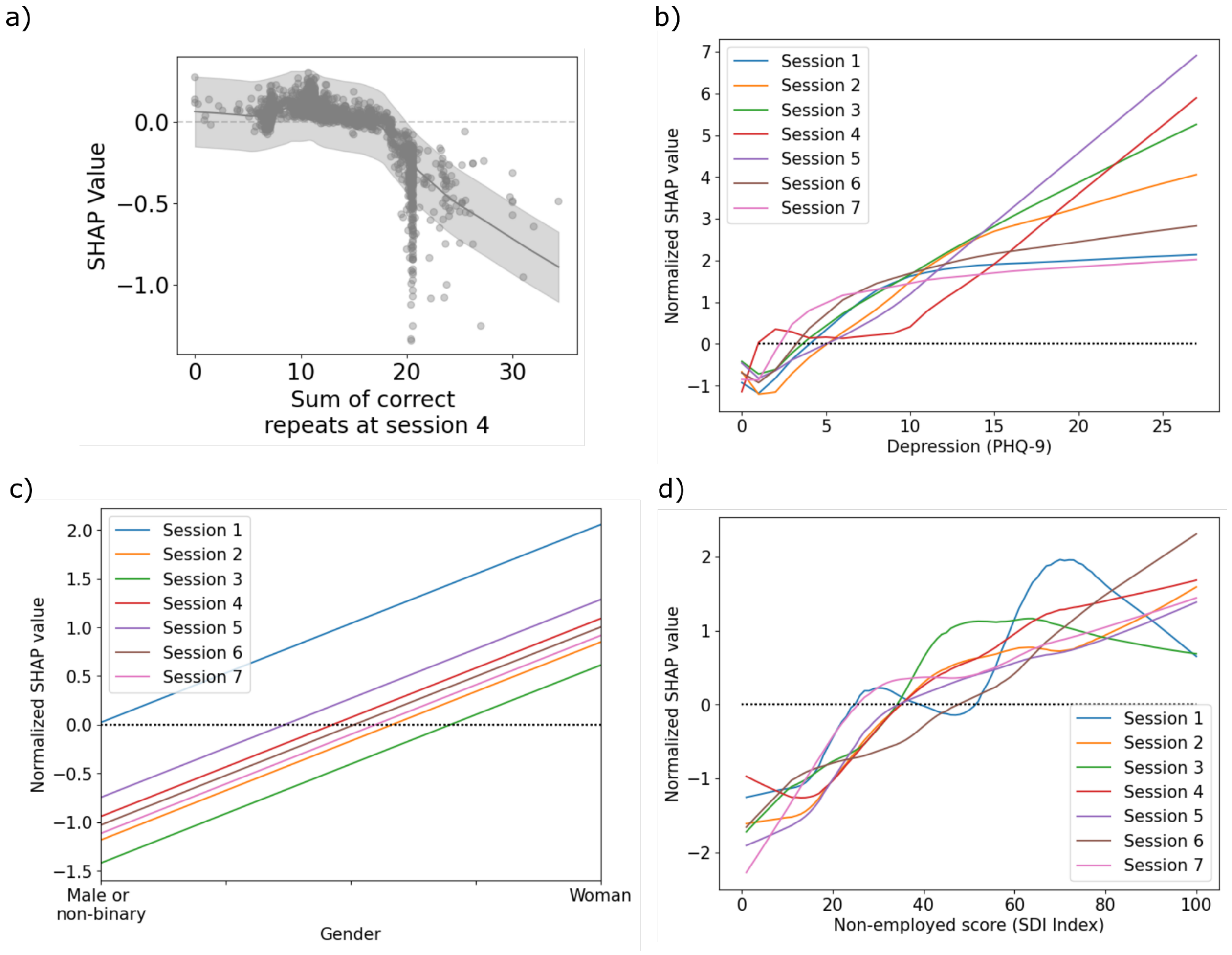

Supplementary Figure 8 - (a) Scatter plots showing the relationship between feature value and SHAP value for sum of correct movements at session 4. The black line and shaded areas represent the mean and SD of the regression line; Regression line fitted to individual SHAP values depicting the relationship between SHAP value and (b) Depression (GAD-7 questionnaire); (c) Gender (d) Non-employed score (from socioeconomic deprivation index). All figures correspond to LightGBM. SHAP values were normalized at each timepoint by dividing by the standard deviation of SHAP values.

Supplementary Table 4 - Model's performance on the test dataset at the 7th session depending on the subgroup domain.

Domain	Sub-group	AUC (7th session)
Gender	Woman (N=1067)	0.70
	Man (N=742)	0.71

Pain	Severe Pain (≥7)	0.72
	Moderate Pain (<7)	0.70
Acuity	Acute (N=329)	0.69
	Chronic (N=1483)	0.69
Social Deprivation index (SDI)	SDI<20 (N=550)	0.69
	SDI≥60 (N=473)	0.68
Age	Age ≥60 (N=318)	0.69
	Age 40-60 (N=1004)	0.72
	Age <40 (N=439)	0.69
